# Limb accelerations during sleep are related to measures of strength, sensation, and spasticity among individuals with spinal cord injury

**DOI:** 10.1186/s12984-022-01090-8

**Published:** 2022-11-03

**Authors:** Stephanie K. Rigot, Michael L. Boninger, Dan Ding, Jennifer L. Collinger, Brad E. Dicianno, Lynn A. Worobey

**Affiliations:** 1 Rehab Neural Engineering Labs, University of Pittsburgh, Pittsburgh, PA USA; 2 Human Engineering Research Laboratories, Veterans Affairs Pittsburgh Healthcare System, Pittsburgh, PA USA; 3 Department of Bioengineering, University of Pittsburgh, Pittsburgh, PA USA; 4 Max Näder Center for Rehabilitation Technologies & Outcomes Research, Shirley Ryan AbilityLab, Chicago, IL USA; 5 Center for Bionic Medicine, Shirley Ryan AbilityLab, Chicago, IL USA; 6 Departments of Rehabilitation Science and Technology, Pittsburgh, PA USA; 7 Departments of Physical Medicine and Rehabilitation, University of Pittsburgh, 3520 Fifth Avenue, Suite 300, 15213 Pittsburgh, PA USA; 8 Departments of Physical Therapy, University of Pittsburgh, Pittsburgh, PA USA

**Keywords:** Functional outcomes, Spinal cord injuries, Motor activity, Physiologic monitoring, Muscle strength, Sensation, Muscle spasticity, Lower extremity, Sleep

## Abstract

**Background:**

To evaluate the relationship between measures of neuromuscular impairment and limb accelerations (LA) collected during sleep among individuals with chronic spinal cord injury (SCI) to provide evidence of construct and concurrent validity for LA as a clinically meaningful measure.

**Methods:**

The strength (lower extremity motor score), sensation (summed lower limb light touch scores), and spasticity (categorized lower limb Modified Ashworth Scale) were measured from 40 adults with chronic (≥ 1 year) SCI. Demographics, pain, sleep quality, and other covariate or confounding factors were measured using self-report questionnaires. Each participant then wore ActiGraph GT9X Link accelerometers on their ankles and wrist continuously for 1–5 days to measure LA from movements during sleep. Regression models with built-in feature selection were used to determine the most relevant LA features and the association to each measure of impairment.

**Results:**

LA features were related to measures of impairment with models explaining 69% and 73% of the variance (R²) in strength and sensation, respectively, and correctly classifying 81.6% (F1-score = 0.814) of the participants into spasticity categories. The most commonly selected LA features included measures of power and frequency (frequency domain), movement direction (correlation between axes), consistency between movements (relation to recent movements), and wavelet energy (signal characteristics). Rolling speed (change in angle of inclination) and movement smoothness (median crossings) were uniquely associated with strength. When LA features were included, an increase of 72% and 222% of the variance was explained for strength and sensation scores, respectively, and there was a 34% increase in spasticity classification accuracy compared to models containing only covariate features such as demographics, sleep quality, and pain.

**Conclusion:**

LA features have shown evidence of having construct and concurrent validity, thus demonstrating that LA are a clinically-relevant measure related to lower limb strength, sensation, and spasticity after SCI. LA may be useful as a more detailed measure of impairment for applications such as clinical prediction models for ambulation.

**Supplementary Information:**

The online version contains supplementary material available at 10.1186/s12984-022-01090-8.

## Background

Wearable accelerometers are commonly used in clinical research as an inexpensive and unobtrusive means of measuring an individual’s movements, mobility, and physical activity, both within and outside of clinical settings. Specifically, for people with spinal cord injury (SCI), accelerometers have been used to assess physical activity and energy expenditure among wheelchair users, [1, 2] assess sleep, [3] predict in-lab versus at-home activities, [4] and count steps during inpatient physical therapy sessions [5].

A variety of time and frequency domain features can be extracted from wearable accelerometer data to provide diverse characteristics of an individual’s movement. We have defined limb accelerations (LA) as features calculated from an individual’s limb movements while asleep at night, which may capture accelerations from periodic limb movements (PLM), spasms, positional shifts, rolling, and turning. These movements are free of biases that daytime activities may introduce secondary to an individual’s occupation, exercise routines, and leisure time activities. LA have previously been shown to provide rich, descriptive information that was related to functional ambulation among a sample with chronic, motor incomplete SCI [6].

While asleep, an individual likely moves mostly subconsciously for comfort, pressure relief, temperature, or in response to other sensations [7, 8]. These movements encompass aspects of sensation to cue the individual to move and strength to perform the movement. For example, it is expected that more complex voluntary or subconscious movements, such as rolling or substantial repositioning movements, would require greater muscle strength than simpler movements, such as subtly moving a limb. Further, someone with better sensation may have more of these complex movements than someone with poorer sensation since they may have increased cues to reposition [7, 8]. Since these more complex movements would likely produce larger accelerations and longer completion times than simpler movements, we would anticipate that individuals with better strength and sensation would have higher amplitude and duration LA than individuals with limited strength and sensation.

Although more intense spasms may result in larger amplitude and duration movements, most spastic movements are relatively small in amplitude and duration [9]. Further, clinical assessments of spasticity, such as the Modified Ashworth Scale (MAS), define the most severe spasticity scores as considerable increase in muscle tone causing movement to be difficult (score of 3) or a rigid joint (score of 4 out of 4) [10]. It is anticipated that an individual with more severe spasticity may experience more resistance to movement and this may result in lower amplitude and shorter duration movements [11, 12]. It has been shown that supine positioning may increase spasticity, thus, spasticity and other involuntary movements may be more prevalent while laying down to sleep at night [13, 14]. Thus, we believe that features of LA measured during sleep can capture the unique attributes of an individual’s movement patterns and are related to clinical measures of strength, sensation, and spasticity among individuals with SCI.

The primary objective of this study was to provide quantitative evidence of validity of LA as a measure of impairment among individuals with SCI. Construct validity is the demonstrated relationship that a measurement is comparable to a different measure assessing a similar concept and unlike dissimilar concepts [15]. Concurrent validity quantifies the relationship between the novel measure and another previously validated measure of the intended construct [15]. We aimed to establish the construct and concurrent validity of LA as a clinically meaningful metric by evaluating the relationship between LA and summative standard clinical measures of lower limb strength, sensation, and spasticity among a population with chronic SCI. We hypothesized that features of LA related to amplitude and duration of movements would be the features most strongly related to each clinical outcome. Further, we anticipated that better strength, sensation, and spasticity would be associated with larger amplitude and longer duration movements. As a supplemental analysis to provide additional evidence of construct validity, we aimed to quantify the unique information provided by LA as compared to models consisting of possible covariate measures such as pain and sleep quality.

## Methods

All participants provided informed consent as approved by the VA Pittsburgh Healthcare System Institutional Review Board. Individuals with chronic (≥ 1 year), motor complete and incomplete SCI were included in this analysis, although individuals with motor complete SCI were recruited in smaller numbers in order to mitigate bias in the impairment score distribution. Participants were excluded if they had a medical diagnosis of a condition that may affect sleep (e.g., sleep apnea), were unable to wear limb accelerometers, or had an injury to the legs that would significantly impair ambulation (e.g., amputation).

Data collection was consistent with methods described in detail in prior work investigating LA among individuals with incomplete SCI [6]. In brief, participants were recruited at adaptive sporting events and from a research registry from 2018 to 2021. Participants completed questionnaires that assessed personal, psychosocial, and environmental factors such as demographics, [16, 17] pain, [18–20] and sleep quality [21]. Participants completed a sleep and activity log for each night of the collection which reported activities that could affect sleep, fatigue and sleep quality ratings, and if the participant considered that night “typical” of how they normally sleep [18, 19, 22–26]. Participants also had their strength, sensation, and spasticity in their upper and lower limbs assessed by one of two trained clinicians following the International Standards for Neurological Classification of Spinal Cord Injury and MAS guidelines, except that all participants were assessed in a seated position. Participants then wore ActiGraph GT9X Link accelerometers for 1–5 days on their bilateral ankles and non-dominant wrist. The duration of collection was limited for some participants by logistical constraints, including the short time frame of the adaptive sporting events.

### Data analysis

#### Input variables: LA and covariates

Only nights that the participant reported as “typical” to how they normally sleep were included in the analysis so that the LA analyzed were most representative of the participant’s normal movements and abilities. Sixty-one LA features were extracted from each ankle movement measured with the accelerometers, and the median and interquartile range of each feature (and maximum of one feature) were calculated across all movements per “typical” night. Using the ankle and wrist accelerometers, 10 additional features were computed per night such as the time asleep and proportions of movements that involved each limb. One final set of features per participant was determined by calculating the median for each feature across all nights of the collection [6]. This resulted in a final set of 133 LA features that captured changes in positioning, movement directions, frequency, smoothness, temporal characteristics, signal stability, and intensity (Supplementary Appendix 1 provides additional descriptions of the features) [6, 27–48].

Since the impairment outcomes were measured cross-sectionally, the measurement of both impairment and LA could be affected by factors such as demographics, pain, sleep quality, exercise, sleep medication, or consumption of caffeine or alcohol (24 covariate features, Supplementary Appendix 2) [16–26]. As a supplemental analysis, we evaluated how much unique variance in impairment was explained by adding selected LA features to models made using only covariates. All features were scaled by the minimum and maximum across participants to a 0–1 scale.

#### Output variables: strength, sensation, and spasticity

Strength was quantified by the lower extremity motor score which sums the manual muscle test motor scores from the L2-S1 myotomes across both lower limbs for a score between 0 (total paralysis) to 50 (normal). Lower limb sensation score was similarly calculated by summing the individual light touch scores from each dermatome across the lower limbs for a total score between 0 (no sensation) and 20 (full sensation) [49].

Spasticity was measured by the MAS for the knee flexors and ankle plantarflexors of both lower limbs. MAS had a skewed distribution in our sample with many participants having no spasticity, only two participants having a MAS score of 3, and no participants having a score of 4 in any of the areas assessed. To address this imbalance in scores and to improve clinical interpretability of lower limb spasticity, the MAS scores were categorized into 3 groups: no, mild, and moderate spasticity. Participants were categorized as “no spasticity” if they had a MAS = 0 for all areas assessed, “mild spasticity” if they had some spasticity (MAS > 0) recorded but all MAS scores were < 2, or “moderate spasticity” if any MAS score was ≥ 2.^10^

#### Analysis models

To select a subset of LA features, the least absolute shrinkage and selection operator (LASSO) implemented with least angle regression (LARS) and multinomial logistic regression with ℓ_1_ regularization algorithms were utilized for the numerical (strength and sensation) and categorical (spasticity) outcomes, respectively. While the LASSO LARS and logistic regression with ℓ_1_ regularization algorithms have much in common with linear regression and logistic regression, respectively, they are preferred in this instance due to their ability to perform feature selection as part of the model building process, making them more efficient for high dimensional data [50–52].

For the supplemental analysis, covariate features were selected using the LASSO LARS and logistic regression with ℓ_1_ regularization algorithms. Baseline performance using only the selected covariate features were then determined using linear and logistic regression models for the numerical and categorical outcomes, respectively. Selected LA features were then added to the covariates to assess the unique variance explained (strength/sensation) or improvement in classification (spasticity) from the addition of LA. All analyses used 10-fold block cross-validation among the randomized participant feature sets in the selection of the optimal features and all available samples in the final regression models.

#### Model evaluation

For the strength and sensation LA models, the primary evaluation metric was R² which represents the variance in the outcome explained by the selected input features. When comparing the explained variance between models, it is important to account for the number of features included in the model, as models with more features likely have greater explained variance. Therefore, the adjusted R², which applies a correction to R² for the number of features in the model, was used as the primary evaluation metric for the supplemental analysis for strength and sensation when comparisons were made between models containing only covariates or with covariates and LA [52]. Statistical significance of the change in the strength and sensation linear regression models when LA features were added to covariates was assessed using the change in the F-statistic and p < 0.05. For both the primary and supplemental analyses, other evaluation metrics included mean absolute error, mean squared error, and root mean squared error. Cohen’s $f$^2^ was used to evaluate effect size from the adjusted R² with 0.02, 0.15, and 0.35 indicative of small, medium, and large effects, respectively [53].

The overall classification accuracy (OCA), precision, recall, and F1-score were used to describe the spasticity model performance. OCA represents the percentage of participants who were correctly classified. Precision represents the accuracy of the true classifications (i.e., positive predictive value) while recall represents the fraction of the correctly identified positive classifications (i.e., true positive rate). The F1-score is the weighted average of precision and recall [54, 55]. The log-likelihood ratio was used to assess for statistically significant change from the addition of LA to the covariates logistic regression models in the supplemental analysis.

#### Evaluation of validity

Construct validity was determined by evaluating the features chosen by each model and the clinical interpretation in relation to the impairment outcome. Further evidence of construct validity was also provided by determining the variance in each impairment outcome that was uniquely explained by LA, in the presence of other factors that could potentially represent similar information to LA or affect the measurement of LA or the impairment outcomes. This provided confirmation that LA are truly a measure of impairment and not simply a surrogate measure of other factors, like sleep quality. Concurrent validity was measured by the variance explained/classification accuracy of the models using LA and the standard clinical assessments.

## Results

### Participants

**Fig. 1:**
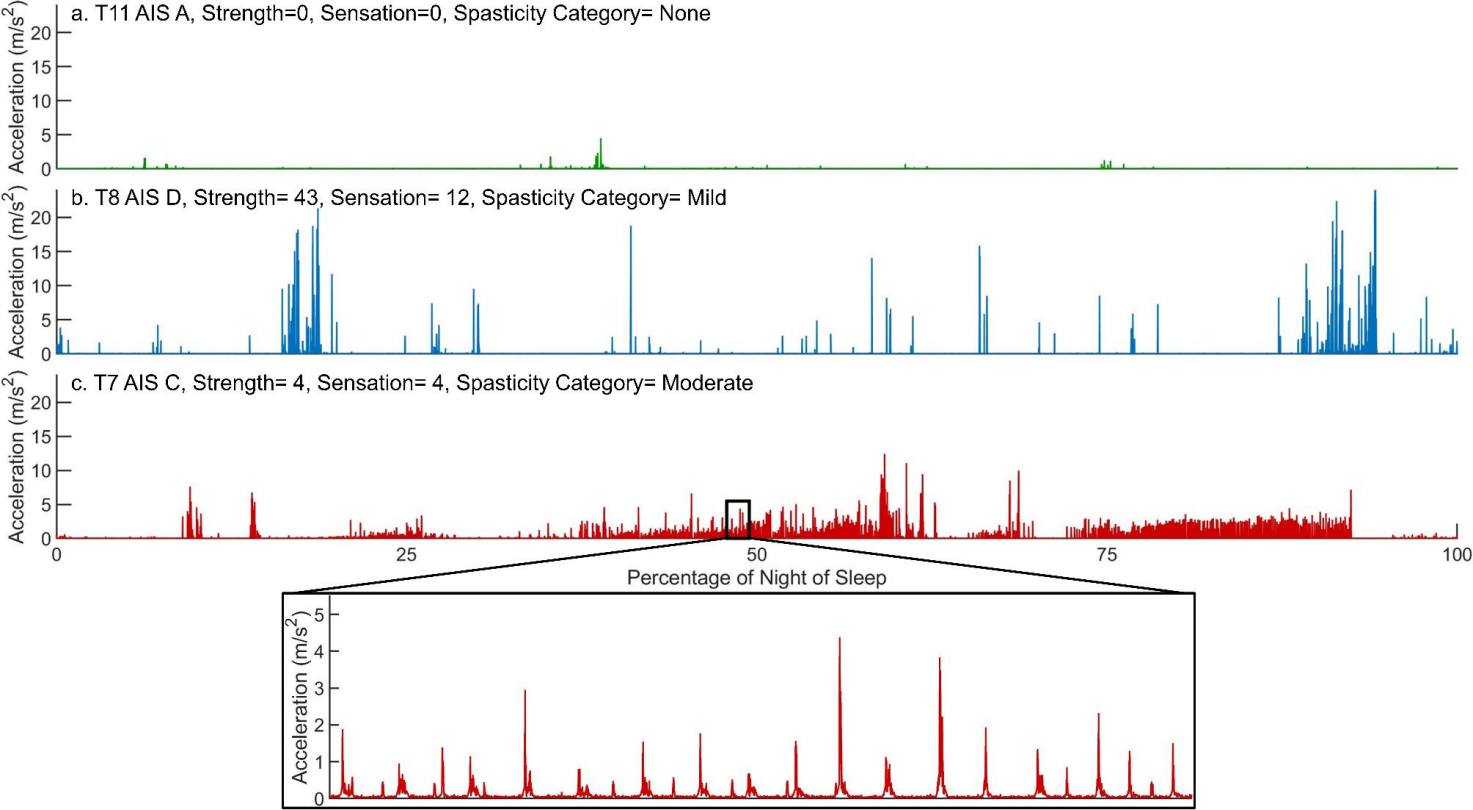
Example of acceleration vector magnitude vs. time plots from one ankle across one night for 3 participants with corresponding demographics and impairment outcomes. Figure 1c includes a zoomed section for additional detail of likely spastic or other involuntary movements

Thirty-six participants with motor incomplete SCI and 13 with motor complete SCI completed the data collection. Eight participants were excluded from the analysis because they self-reported that they had no “typical” nights recorded during the collection period. One additional participant was excluded because the accelerometers were likely removed overnight. Data collection was completed for two participants before the spasticity measures were added to the study, so the spasticity analysis had 38 total participants included, while the strength and sensation analyses had 40 participants. A post hoc power analysis showed that > 88% power was achieved for all linear regression models given the sample size, number of predictors, α = 0.1, and effect size ($f$^2^) [56]. Participants were primarily male, non-Hispanic/Latino White, Veterans with paraplegia who used a manual wheelchair as their primary mode of mobility (Table 1). Examples of ankle acceleration plots are shown in Fig. 1.

**Table 1:** Participant demographics and impairment outcomes

Categorical Demographics	Motor Incomplete n (% of group)	Motor Complete n (% of group)	Total N (%)
Sex			
Female	4 (13.3)	2 (20.0)	6 (15.0)
Male	26 (86.7)	8 (80.0)	34 (85.0)
Race/Ethnicity			
Non-Hispanic/Latino White	14 (46.7)	6 (60.0)	20 (50.0)
Non-Hispanic/Latino Black	10 (33.3)	3 (30.0)	13 (32.5)
Non-Hispanic/Latino Other Race	3 (10.0)	0 (0.0)	3 (7.5)
Hispanic/Latino (Any Race)	3 (10.0)	1 (10.0)	4 (10.0)
Veteran			
Not Veteran	5 (16.7)	0 (0.0)	5 (12.5)
Veteran	25 (83.3)	10 (100)	35 (87.5)
Annual Household Income			
<$25,000	9 (30.0)	2 (20.0)	11 (27.5)
$25,000-$49,999	3 (10.0)	5 (50.0)	8 (20.0)
$50,000-$74,999	5 (16.7)	2 (20.0)	7 (17.5)
≥$75,000	9 (30.0)	1 (10.0)	5 (12.5)
Decline to Answer or Unknown	4 (13.3)	0 (0.0)	4 (10.0)
Education			
High School Diploma/GED	17 (56.7)	2 (20.0)	19 (47.5)
Associate’s Degree	7 (23.3)	4 (40.0)	11 (27.5)
Bachelor’s Degree	4 (13.3)	2 (20.0)	6 (15.5)
Graduate Degree	2 (6.7)	2 (20.0)	4 (10.0)
SCI Injury Level			
Paraplegia	19 (63.3)	9 (90.0)	28 (70.0)
Tetraplegia	11 (36.7)	1 (10.0)	12 (30.0)
SCI American Spinal Injury Association Impairment Scale (AIS) Classification (Calculated)			
A	0 (0.0)	6 (60.0)	6 (15.0)
B	0 (0.0)	4 (40.0)	4 (10.0)
C	15 (50.0)	0 (0.0)	15 (37.5)
D	15 (50.0)	0 (0.0)	15 (37.5)
Data Collection Location			
Local	8 (26.7)	0 (0.0)	8 (20.0)
Adapted Sporting Event	22 (73.3)	10 (100)	32 (80.0)
Primary Mode of Mobility			
Walk	5 (16.7)	0 (0.0)	5 (12.5)
Manual Wheelchair	20 (73.5)	8 (80.0)	28 (70.0)
Power Wheelchair/Scooter	4 (8.8)	2 (20.0)	6 (15.0)
Equally Walk and Wheel	1 (3.3)	0 (0.0)	1 (2.5)
**Categorical Impairment Outcomes**	**Motor Incomplete** n (% of group)	**Motor Complete** n (% of group)	**Total** N (%)
Spasticity (Lower Limb Categorized MAS)			
No Spasticity (MAS = 0)	13 (46.4)	2 (20.0)	15 (39.5)
Mild Spasticity (MAS all < 2)	9 (32.1)	5 (20.0)	14 (36.8)
Moderate Spasticity (≥ 1 area MAS ≥ 2)	6 (21.4)	3 (30.0)	9 (23.7)
**Numerical Demographics**	**Motor Incomplete** Mean ± SD (Range)	**Motor Complete** Mean ± SD (Range)	**Total** Mean ± SD (Range)
Age	54.0 ± 10.5 (25–70)	52.9 ± 14.2 (34–77)	53.7 ± 11.4 (25–77)
Body Mass Index (BMI)	28.2 ± 5.4 (18.5–38.7)	24.4 ± 3.7 (18.7–30.4)	27.2 ± 5.2 (18.5–38.7)
Years Since Injury	18.8 ± 12.5 (3.0-48.7)	16.0 ± 9.5 (5.6–28.9)	18.1 ± 11.8 (3-48.7)
Number of Nights Collected	2.6 ± 1.3 (1–5)	2.0 ± 0 (2–2)	2.5 ± 1.2 (1–5)
Number of Typical Nights	2.0 ± 1.1 (1–5)	1.9 ± 0.3 (1–2)	2.0 ± 1.0 (1–5)
**Numerical Impairment Outcomes**	**Motor Incomplete** Mean ± SD (Range)	**Motor Complete** Mean ± SD (Range)	**Total** Mean ± SD (Range)
Strength (Lower Extremity Motor Score)	26.9 ± 15.0 (2–49)	0.0 ± 0.0	8.2 ± 8.1 (0–49)
Sensation (Lower Limb Light Touch)	10.9 ± 6.5 (0–20)	3.8 ± 6.6 (0–20)	9.1 ± 7.2 (0–20)

### Strength

Sixteen LA features were selected which explained 68.7% of the variance in lower limb strength (Table 2). The features with the greatest association with higher strength scores were larger variations in energy (Wave Approx- IQR), fewer variations in the similarity between recent movements (Num Cross Corr Peaks- IQR), greater variation in local dynamic stability (variations in the response to perturbations, Lyapunov Exp- IQR) and faster rotational movements (Angle Rate Change- Med, Tables 3 and 4). When LA features were combined with covariates, an additional 35.5% of the variance in strength could be explained (adjusted R^2^ = 0.847, 72% increase, p = 0.021), as compared to the model with only covariates (Supplementary Appendices 3 and 4).

**Table 2:** Strength, sensation, and spasticity model results

Impairment Model	Number of Features Selected	R²	Adjusted R²	$f$ ^2^	Mean Absolute Error	Mean Squared Error	Root Mean Squared Error
Strength	16	0.687	0.469	0.88	9.17	93.56	9.67
Sensation	15	0.733	0.566	1.31	2.91	13.32	3.65
		**Predicted Spasticity Category**					
**Actual Spasticity Category**	**Number of Features Selected**	**No Spasticity**	**Mild Spasticity**	**Moderate Spasticity**	**F1-Score**	**Precision**	**Recall**
No Spasticity	10	**14**	1	0	0.848	0.778	0.933
Mild Spasticity	7	3	**11**	0	0.786	0.786	0.786
Moderate Spasticity	10	1	2	**6**	0.800	1.000	0.667
Macro Average					0.811	0.854	0.795
**Weighted Average**					**0.814**	**0.833**	**0.816**

**Table 3:** Number (percentage) of selected LA features for each impairment outcome by category, with darker shading representing a higher proportion of features selected

LA Feature Category	Strength	Sensation	Spasticity		
			None	Mild	Moderate
Change in angle of inclination (Ang)	1 (6.3%)	0 (0%)	0 (0%)	0 (0%)	0 (0%)
Correlation coefficients between axes (CC)	2 (12.5%)	2 (13.3%)	1 (10.0%)	1 (14.3%)	1 (10.0%)
Change in gravitational acceleration (CGA)	0 (0%)	1 (6.7%)	0 (0%)	1 (14.3%)	0 (0%)
Frequency domain (Freq)	3 (18.8%)	5 (33.3%)	2 (20.0%)	2 (28.6%)	3 (30.0%)
Limb movement percentages	0 (0%)	0 (0%)	0 (0%)	0 (0%)	0 (0%)
Median crossings (MC)	1 (6.3%)	0 (0%)	0 (0%)	0 (0%)	0 (0%)
Periodic limb movements (PLM)	1 (6.3%)	1 (6.7%)	1 (10.0%)	0 (0%)	1 (10.0%)
Relationship to recent movements (RRM)	4 (25.0%)	3 (20.0%)	3 (30.0%)	2 (28.6%)	0 (0%)
Signal characteristics (SC)	2 (12.5%)	2 (13.3%)	2 (20.0%)	0 (0%)	3 (30.0%)
Statistical (Stat)	1 (6.3%)	0 (0%)	1 (10.0%)	0 (0%)	1 (10.0%)
Timing (Time)	1 (6.3%)	1 (6.7%)	0 (0%)	1 (14.3%)	1 (10.0%)
Velocity and distance	0 (0%)	0 (0%)	0 (0%)	0 (0%)	0 (0%)
Total LA features selected	16	15	10	7	10

**Table 4:** LA features included in the strength, sensation, and spasticity models, sorted by the absolute value of the coefficient

Strength (16 features)						Sensation (15 features)					
Feature Category		Feature Name			Coeff	Feature Category	Feature Name			Coeff	
SC		Wave Approx- IQR			20.99	RRM	Time Since Prev- IQR			-9.62	
RRM		Num Cross Corr Peaks- IQR			-19.81	CC	Corr YZ- Med			8.34	
SC		Lyapunov Exp- IQR			13.90	Freq	Dom Freq 1- Med			-8.10	
Ang		Angle Rate Change- Med			13.22	RRM	Num Cross Cov Peaks- IQR			-7.15	
Freq		Power Dom Freq 1/Total- IQR			11.64	Time	Time Asleep			6.92	
CC		Corr XY- IQR			8.83	SC	Wave Entropy- IQR			5.67	
RRM		Move Next 90s- Med			8.67	RRM	Num Cross Corr Peaks- IQR			-4.85	
Freq		Med Freq- Med			-6.08	Freq	Power Dom Freq 2- Med			-3.72	
RRM		Max Cross Cov- IQR			-5.74	Freq	Dom Freq 1- IQR			3.48	
CC		Corr XY- Med			-5.73	CGA	Grav Change Y- IQR			3.17	
PLM		PLM %			5.42	Freq	Power Dom Freq 1/Total- IQR			2.08	
RRM		Time Since Prev- IQR			-4.61	Freq	Mean Freq- IQR			1.18	
Time		Time Asleep			2.09	SC	Lyapunov Exp- Med			1.12	
Freq		Mean Freq- IQR			0.70	CC	Corr XZ- IQR			-0.59	
MC		Num Med Crossings Norm- Med			-0.64	PLM	PLM Index			0.11	
Stat		Skewness- Med			-0.51						
**No Spasticity** (10 features)				**Mild Spasticity** (7 features)				**Moderate Spasticity** (10 features)			
Feature Category	Feature Name		Coeff	Feature Category	Feature Name		Coeff	Feature Category	Feature Name		Coeff
CC	Corr YZ- Med		-0.95	Time	Time Asleep		1.38	SC	Wave Entropy- IQR		-2.26
Stat	Skewness- IQR		0.93	RRM	Num Cross Cov Peaks- IQR		-0.74	Time	Move/hour		1.70
Freq	Power Dom Freq 2- Med		-0.62	Freq	Power Dom Freq 1- IQR		0.48	Stat	Skewness- IQR		-1.03
RRM	Close Cross Cov Peak- IQR		0.59	CGA	Grav Change Z- IQR		-0.37	SC	Wave Energy 2- Med		-0.91
RRM	Close Cross Corr Peak- IQR		0.58	CC	Corr XY- Med		-0.27	Freq	Med Freq- Med		0.60
SC	Wave Energy 2- Med		0.54	RRM	Dom Freq Last 90s- IQR		-0.06	CC	Corr YZ- Med		0.38
Freq	Dom Freq 2- IQR		0.39	Freq	Dom Freq 1- Med		-0.04	SC	Wave Approx- IQR		-0.27
PLM	PLM %		-0.34					Freq	Med Freq- IQR		0.17
SC	Wave Approx- IQR		0.15					PLM	PLM Index		0.17
RRM	Num Cross Cov Peaks- IQR		0.06					Freq	Dom Freq 1- Med		0.04

Abbreviations: Ang = Change in angle of inclination, CC = Correlation coefficients between axes, CGA = Change in gravitation acceleration, Coeff = Model Coefficient, Freq = Frequency domain, MC = Median crossings, Med = Median, PLM = Periodic limb movements, RRM = Relationship to recent movements, SC = Signal characteristics, Stat = Statistical, Time = Timing

### Sensation

A model containing 15 LA features explained 73.3% of the variance in lower limb sensation. Having a less variable time between movements (Time Since Prev- IQR), more consistent movement directions (Corr YZ- Med), and lower frequency movements (Dom Freq 1- Med) were most strongly associated with more intact sensation. When added to covariates, LA explained an additional 49.2% of the variance in sensation (adjusted R^2^ = 0.714, 222% increase, p = 0.001).

### Spasticity

Spasticity categories had an OCA of 81.6% using 7–10 selected LA features (weighted average F1-Score = 0.814). No participants were falsely classified as having moderate spasticity (precision = 1), but the highest recall (0.933) was for the no spasticity category, indicating that those without spasticity were most likely to be correctly classified. The features most associated with having no spasticity included moving in less consistent directions (Corr YZ- Med), more variable movement symmetry (Skewness- IQR), less power at the second dominant frequency (Power Dom Freq 2- Med), and more variable recent movements (Close Cross Cov/Corr Peak- IQR). LA features associated with moderate spasticity include less variable movement entropy (Wave Entropy- IQR), more movements per hour (Move/hour), less variable symmetry of movements (Skewness- IQR), and lower and less variable energy (Fig. 1c). When combined, the LA + covariates model achieved 89.5% accuracy in classifying spasticity categories, including an increase in the weighted average F1-score of 0.186 (26% increase, p < 0.001, p = 0.275, and p < 0.001 for no, mild, and moderate spasticity models, respectively) as compared to the model using only covariates.

## Discussion

We have provided evidence of construct and concurrent validity for LA as a measure of impairment by demonstrating that regression models consisting of only LA features were able to explain the majority of the variance in strength and sensation and correctly classify the majority of participants into spasticity categories. Since LA features are continuous, LA may provide variability and detailed information about impairment that clinical measures currently lack and, thus, may be useful in providing increased resolution compared to existing measures. Further, in the supplemental analysis, LA accounted for additional variance beyond what can be attributed to covariates alone, thus, supporting the construct validity that LA features uniquely capture aspects associated with each impairment outcome and not related measures like sleep quality.

It was hypothesized that LA features such as those measuring amplitude and duration of movements would be most related to the measures of impairment with larger amplitudes and durations being associated with better impairment outcomes, which had mixed support from the findings. Movement duration was not selected for any of the models, and therefore was not directly among the most important LA features in relation to each outcome. However, other features that may indirectly contain movement duration information, such as the percentage of movements that meet the criteria for PLM (PLM %) and PLM per hour (PLM Index), were selected in nearly all models. By definition, PLM must be short duration movements that occur in series [41, 44]. Therefore, having a higher percentage of movements that meet the criteria for PLM being related to greater strength, better sensation, and less spasticity provides support that movement duration, in combination with the other characteristics that define a movement as part of a PLM series, may be an important aspect of LA in relation to impairment.

As shown in Tables 3 and 4, features evaluating the spectral power in the frequency domain and wavelet energy bands (signal characteristics such as Power Dom Freq 2- Med, Power Dom Freq 1/Total- IQR, Wave Approx- IQR) of movements were often some of the most strongly related features to each measure of impairment and were selected for each impairment outcome. Both the statistical and frequency domain features consist of similar information about the intensity of movements, but the statistical features are with respect to time while features like power and energy are with respect to frequency or both time and frequency. Therefore, it makes intuitive sense that higher power and energy movements may be associated with greater strength and less severe spasticity. Likewise, more impaired sensation was associated with higher and less variable frequency (Dom Freq 1- Med/IQR) and more powerful movements (Power Dom Freq 2- Med) suggesting a lack of motor control to vary and regulate movements based upon sensory feedback. Therefore, the hypothesis that larger amplitude movements would be associated with improved outcomes was indirectly supported for strength and spasticity. The hypothesis was not supported for sensation since higher power movements with lower frequency were associated with poorer sensation. Similar features have also been found to be related to lower limb rehabilitation [36] and gait among various populations, [34] further indicating the clinical relevance of these measures.

Movement consistency was found to be related to all impairment outcomes through the consistency of movement directions (correlation coefficients between axes) and consistency between movements (PLM, relationship to recent movements, timing). For example, more consistently timed movements were related to greater strength (Num Cross Corr Peaks- IQR, Time Since Prev- IQR), better sensation (Time Since Prev- IQR, Num Cross Cov/Corr Peaks- IQR, PLM Index), and less spasticity (No Spasticity: Close Cross Cov/Corr Peak- IQR, PLM %; Moderate Spasticity: Move/hour, PLM Index). Additionally, moving in a larger variety of directions was associated with greater strength (Corr XY- IQR/Med) and less spasticity (Corr YZ- Med), but worse sensation (Corr YZ- Med, Corr XZ- IQR). Since moving in a variety of directions requires muscle activation from a greater number of locations, it makes sense that moving in more directions was related to greater strength. Additionally, it is logical to infer that participants with more frequent, consistent, repetitive movements may have more severe spasticity (or related involuntary movements such as myoclonus or PLM) while those with more variable, less consistent movements have little to no spasticity. Since about 40% of individuals with SCI may experience problematic spasms that affect their sleep, [57, 58] LA may be an unobtrusive way to evaluate spasticity and the effects of treatment.

Additional measures of movement consistency that were related to greater strength include having a wider range of responses to perturbations (higher Lyapunov Exp- IQR), smoother movements (lower Num Med Crossings Norm- Med), and more negatively skewed movements (lower Skewness- Med). These findings are supported by previous studies that have shown the Lyapunov exponent to be related to improvements in lower limb rehabilitation [36] and ambulation [6] and that healthy controls generally had smoother movements and more negative skewness than individuals with Parkinson’s disease [34].

Limb movement percentages and velocity and distance features were not selected for any impairment outcomes. While velocity and distance information may be better represented by similar features such as those in the frequency domain, limb movement percentages represented unique information that may not be as meaningfully related to strength, sensation, or spasticity.

Having faster rotational movements (i.e., rolling, Angle Rate Change- Med) and smoother movements (Num Med Crossings Norm- Med) were related to greater strength, but were not among the selected features for sensation or spasticity. The ability for certain categories of LA to be related to some measures of impairment but not others, demonstrates the vast amount of diverse information that LA can detect which may provide increased resolution about an individual’s strength, sensation, and spasticity than current clinical measures. This may be particularly useful in the context of clinical prediction rules, as models using common clinical assessments may be inadequate to accurately predicting long-term functional ambulation among those with incomplete SCI [16, 59–61].

Our previous work found that LA improved the classification accuracy of categories of functional ambulation among those with motor incomplete SCI [6]. This further supports the validity of LA and demonstrates that LA likely contain richer information than clinical measures of impairment alone. Future studies should evaluate other lower limb functional tasks and how LA can be utilized for outcome prediction in a longitudinal sample with acute SCI.

Although LA features were specifically extracted to be clinically meaningful individually, they provide the most beneficial and comprehensive information when interpreted together [34]. Since all LA features are calculated using the same data set with minimal computational time to extract many features, one can obtain a versatile set of detailed features related to impairment with minimal collection burden.

### Limitations

Although only nights “typical” to how the participant normally sleeps were included in the analysis, this measure was self-reported by the participants and it is possible that even during typical nights, LA were affected by unusual sleep patterns. Factors that may affect the LA data collection such as exercising, consuming alcohol, and daily and overall sleep quality were included in the initial covariate models to ensure that these factors were accounted for in the supplemental analysis. Participants were excluded if they self-reported a medical diagnosis of a condition that affects sleep. Given the high proportion of individuals with chronic SCI who have sleep-disordered breathing and the demographics of the sample, [62, 63]. It is possible that participants were included in the sample that had an undiagnosed sleep disorder. Further research should examine the differences in LA between typical and atypical nights, as well as the effect of sleep disorders on LA.

Although participants were asked to report any medications that they took that may affect sleep, details such as when these medications were taken and if participants used any medications to decrease spasticity were not explicitly recorded. The sample population had a lower proportion of participants with moderate to severe spasticity than expected. Although the results support LA being associated with up to moderate spasticity, future studies should assess the relationship between LA and severe spasticity and the effect of antispasmodic medications on LA.

It is possible that the demographics of the sample are not sufficiently representative of the general SCI population and differences in demographic factors could have affected the presented results. Additional analyses using a larger, representative sample of individuals with SCI should be explored to verify the current findings. Differences in LA based upon participant demographics, such as sex and completeness of injury, should be also explored.

The clinical measures of strength, sensation and impairment have limitations and are not an ideal gold standard for comparison [10, 64–66]. However, using summed measures of strength and sensation over the whole lower limbs, a categorized measure of lower limb spasticity, and only two clinicians for all assessments should minimize the effect of the limitations in reliability and responsiveness seen in the individual measurements [66–68].

For prediction models, it is critical that the model is assessed using a separate, unseen test set to avoid results that appear favorable, but perform poorly in practice. However, we do not intend to use LA as a predictor of impairment, as this is not clinically useful nor a goal of the current analysis. Thus, holding out a separate test set of samples or using a computationally intensive analysis such as nested cross-validation to assess the model performance on unseen data was not deemed necessary. Therefore, the results from this analysis are effective for estimating the relationship between LA and measures of impairment in our sample and demonstrating the validity of LA as a clinical measure. If prediction is a goal of a future analysis, then utilization of a larger sample and a strict validation method with an unseen test set would be required.

It is possible that the LASSO LARS and multinomial logistic regression with ℓ_1_ regularization algorithms that were used for feature selection were affected by noise in the input features and resulted in suboptimal feature selection or selection of redundant features. The use of 10-fold cross-validation was intended to minimize this possibility. Further, additional steps were taken (targeted participant recruitment, collection of multiple nights when possible, etc.) to minimize the bias in the data and maximize the generalizability of the findings.

## Conclusion

Finding that LA measured during sleep is uniquely related to standard clinical measures of strength, sensation, and spasticity has provided evidence of construct and concurrent validity among a sample with chronic SCI. This demonstrates that features derived from LA are clinically meaningful metrics related to neuromuscular impairment that could be useful in many future applications including clinical prediction rules for ambulation after an acute SCI.

## Electronic supplementary material

Below is the link to the electronic supplementary material.

Supplementary Material 1: LA Features v2_ESM.docx

Supplementary Material 2: Covariates included in the analysis.docx

Supplementary Material 3: Covariates Model Results_ESM.docx

Supplementary Material 4: LA Covariates Selected Features_ESM.docx

## Data Availability

The datasets used and/or analyzed during the current study are available from the corresponding author on reasonable request.

## Abbreviations

LA: Limb accelerations.
OCA: Overall classification accuracy.
PLM: Periodic limb movement.
MAS: Modified Ashworth Scale.
SCI: Spinal cord injury.
