# Supplementary material for: Limb accelerations during sleep are related to measures of strength, sensation, and spasticity among individuals with spinal cord injury
Source: J Neuroeng Rehabil. 2022 Nov 3;19:118. doi: 10.1186/s12984-022-01090-8 (PMC9635075; doi:10.1186/s12984-022-01090-8)
Supplement: Supplementary file 1 — Supplementary Material 1: LA Features v2_ESM.docx [file 12984_2022_1090_MOESM1_ESM.docx]

Supplementary Appendix 1: Descriptions and abbreviation key of LA features extracted and included in the analysis. “Feature Short Name” is the name used to reference the LA feature. All features are calculated per ankle accelerometer and per movement and combined by taking the median and IQR across all movements per night (unless otherwise noted) and then the median across all nights in the collection.

| Feature Category | Feature | Feature Short Name | Feature Description | Larger value indicates… |
| --- | --- | --- | --- | --- |
| Change in angle of inclination ^27-30^ | Net | Angle Net Change | Change from the start and end position angles | Larger net change in body position |
|  | Rate | Angle Rate Change | Total change in position divided by duration of movement | Faster positional changes |
|  | Total | Angle Total Change | Total change in position throughout movement | Larger positional changes |
| Correlation coefficients between axes ^31-33^ | x-y | Corr XY | Describes the relationship between axes during a movement | Movements occurring in more consistent directions |
|  | x-z | Corr XZ |  |  |
|  | y-z | Corr YZ |  |  |
| Change in gravitational acceleration ^30, 31, 33^ | X | Grav Change X | Describes changes in body positions with respect to the gravitational (DC) vector | Larger change in proportion of gravitational vector in each direction |
|  | Y | Grav Change Y |  |  |
|  | Z | Grav Change Z |  |  |
| Frequency domain ^31, 34-42^ | Bandwidth | Bandwidth | Range of frequencies that contain 95% of the total power | Movements use a larger range of frequencies |
|  | Centroid frequency | Centroid Freq | Frequency that divides the spectral power distribution into two equal parts | Higher frequency movements |
|  | 1st dominant frequency | Dom Freq 1 | Frequency at maximum spectral power |  |
|  | 2nd dominant frequency | Dom Freq 2 | Frequency at 2nd highest peak of spectral power |  |
|  | Dominant frequency in low frequency range | Dom Low Freq | Isolating just frequencies most likely to contain human movements (0.6-2.5 Hz) |  |
|  | Mean/Median frequency | Mean Freq, Med Freq | Estimate of mean/median normalized frequency |  |
|  | Ratio power at dominant frequency to total | Power Dom Freq 1/ Total | Proportion of the total power that occurs at the dominant frequency | Higher proportion of the total energy occurred at the dominant frequency |
|  | Power at 1st dominant frequency | Power Dom Freq 1 | Maximum power | More powerful movements |
|  | Power at 2nd dominant frequency | Power Dom Freq 2 | Max power at 2nd highest peak of power spectrum |  |
|  | Power at dominant frequency in low frequency range | Power Dom Low Freq | Power at frequencies most likely to contain human movement (0.6 - 2.5 Hz) |  |
|  | Ratio of high frequency power to total | Power High Freq/Total | Proportion of power that may likely be noise and not produced from movement (> 3.5 Hz) | More noisy movement signals |
|  | Total power | Power Total | Area under the power spectral density curve | Higher energy, more powerful movements |

Supplementary Appendix 1 Continued

| Feature Category | Feature | Feature Short Name | Feature Description | Larger value indicates… |
| --- | --- | --- | --- | --- |
| Limb movement percentages | * Bilateral ankle | Bilat Ankle % | Proportion of movements where both ankles are moving simultaneously | Possible increased synergistic movements or lack of motor control to isolate limbs independently |
|  | * Unilateral ankle | Unilat Ankle % | Proportion of movements where only the unilateral ankle is moving | Possible increased spasticity or strength on one lower limb |
|  | * Wrist and bilateral ankles | Whole Body % | Proportion of whole body movements (i.e., rolling) | More whole body movements |
|  | * Wrist and unilateral ankle | Wrist Ankle % | Proportion of movements where both the ankle and wrist are moving simultaneously | Possible increased strength on one side of the body |
| Median crossings ^37, 38, 43^ | Number of crossings | Num Med Crossings,  Num Med Crossings Norm | Measure of movement smoothness | Less smooth movements |
| Periodic limb movements (PLM) ^41, 44^ | * Number of series | Num PLM Norm | Total number of movements meeting approximate criteria to be defined as PLM (movement duration 0.5-10 seconds, series of 4 or more movements in 5-90 second intervals) | More series of short, repetitive movements (likely spastic or PLM) |
|  | * Index | PLM Index | Total number of movements meeting approximate criteria to be defined as PLM divided by the number of hours asleep (>15 events/hour is indicative of possible dysfunction) |  |
|  | * Percentage of movements | PLM % | Percent of all movements occurring during the night that could be classified as PLM | Higher proportion of total movements are occurring in short, repetitive series (likely spastic or PLM) |
| Relationship to recent movements ^32-35, 37^ | Dominant frequency in last 90s | Dom Freq Last 90s | Frequency of recent movement series | More movements occurring in series |
|  | Cross-correlation/ covariances in last 90s | Close Cross Corr/Cov Peak,  Max Cross Corr/Cov,  Mean Cross Corr/Cov Peaks  Num Cross Corr/Cov Peaks | Similarity between recent movement (calculates: closest peak values, maximum value, mean peak values, number of peaks) | More similar/repetitive recent movements |
|  | Number of movements in next/last 90s | Move Next/Last 90s | Quantifies if movement occurred as part of a series | More short, frequent movements are part of a series |
|  | Time since previous movement | Time Since Prev | Seconds since last movement ended | More sparse movements |

Supplementary Appendix 1 Continued

| Feature Category | Feature | Feature Short Name | Feature Description | Larger value indicates… |
| --- | --- | --- | --- | --- |
| Signal characteristics ^32, 34-36^ | Entropy rate | Entropy Rate | Measure of signal regularity | More regular movement accelerations (samples within a movement are more related and less random) |
|  | Lempel-Ziv complexity | Lempel-Ziv Comp | Measure of complexity-probability | Less predictable, more complex accelerations |
|  | Maximum Lyapunov exponent | Lyapunov Exp | Measure of local dynamic stability (sensitivity to perturbations) | More chaos/divergent accelerations from intended path, less stable |
|  | Wavelet energy | Wave Approx,  Wave Energy 1, Wave Energy 2, Wave Energy 3 | Approximation, 1^st^ - 3^rd^ details of the wavelet transform to evaluate the relative energy in each time-frequency band | Higher energy concentration |
|  | Wavelet entropy | Wave Entropy | Measure of signal disorder | More random process/more disorder |
| Statistical ^33-35, 37-41^ | Area under the curve | AUC Acc,  AUC Acc Norm | Total change in velocity | Larger total change in speed |

|  | Signal magnitude area | SMA Acc |  |  |
| --- | --- | --- | --- | --- |
|  | Duration † | Duration | How long each movement lasts | Longer movements |
|  | Kurtosis | Kurtosis | Describes weight of the movement's tails relative to the center of the movement | More widely spread accelerations (less distinguishable max value) |
|  | Maximum to RMS | Max-RMS Acc | Measure of movement smoothness | More jerky movements |
|  | Maximum | Max Acc | Measure of acceleration magnitude | Larger changes in speed |
|  | Range | Range Acc | Maximum to minimum acceleration |  |
|  | Median | Med Acc | Median of acceleration magnitude | Larger magnitude movements |
|  | Root mean square (RMS) | RMS Acc | RMS of acceleration magnitude |  |
|  | SD | SD Acc | Variability of acceleration magnitude | Larger variation within movements |
|  | Skewness | Skewness | Describes the symmetry of the temporal spread of a movement | Positively skewed movements: largest acceleration for movements occur early and then there are longer slow-down periods |
| Timing ^33, 44, 45^ | * Number of movements | Move/night, Move/hour | Number of movements per night/hour | More movements |
|  | When movements occurred in night | Start Move %,  End Move % | Determine if movement are clustered in a certain portion of the night or well distributed | Movements occur later in the night |
|  | * Time asleep | Time Asleep | Hours asleep | Longer time asleep |
| Velocity and distance ^28, 45^ | Median velocity | Med Vel | Movement speed | Faster movements |
|  | RMS velocity | RMS Vel |  |  |
|  | Total distance | Total Dist | Total meters traveled | Further distance moved |
| * Indicates a feature calculated per night  † Indicates maximum across all movements per night also calculated | | | | |
