# Supplementary material for: Limb accelerations during sleep are related to measures of strength, sensation, and spasticity among individuals with spinal cord injury
Source: J Neuroeng Rehabil. 2022 Nov 3;19:118. doi: 10.1186/s12984-022-01090-8 (PMC9635075; doi:10.1186/s12984-022-01090-8)
Supplement: Supplementary file 2 — Supplementary Material 2: Covariates included in the analysis.docx [file 12984_2022_1090_MOESM2_ESM.docx]

Supplementary Appendix 2: Covariates included in the analysis

| Covariate Category | Covariates Features | Covariate Feature Name | Covariate Description | Source |
| --- | --- | --- | --- | --- |
| Demographics ^16, 17^ | Age | Age | In years | Demographics questionnaire |
|  | Years Since Injury | Years Since Injury |  |  |
|  | Body Mass Index | BMI | In kg/m^2^ |  |
|  | Sex | Sex | Male or female |  |
| Pain ^18-20^ | Average pain intensity | Ave Pain Intensity | 0 (no pain) to 10 (extreme pain) | International SCI Pain Basic Data Set Version 1.1 |
|  | If pain present | Pain Present | 0 (no pain present) or 1 (Pain present) |  |
|  | Number of pain locations | Number of Pain Locations | 0 (no pain) to 5 (≥ 5 locations) |  |
|  | Pain interference with sleep | Pain Interference: Sleep | 0 (no interference) to 6 (extreme interference) |  |
|  | Pain Domain Score | SF-36: Pain | Bodily pain and pain interference from 0 to 100 with higher scores indicating less pain | Medical Outcomes Study 36-Item Short-Form Health Survey (SF-36) |
| Sleep Quality ^21^ | Components:   - Daytime dysfunction - Disturbances - Duration - Efficiency - Latency - Sleep quality | PSQI: Daytime Dysfunction  PSQI: Sleep Disturbance  PSQI: Sleep Duration  PSQI: Sleep Efficiency  PSQI: Sleep Latency  PSQI: Sleep Quality | Sleep quality over the previous month in each domain on a scale from 0 to 3 with higher scores indicating worse sleep | Pittsburgh Sleep Quality Index (PSQI) |
|  | Global score | PSQI: Global Score | Sum of 7 components |  |
|  | Poor sleep quality | PSQI: Poor Sleep Quality | Global score >5 (poor sleep quality) or ≤5 |  |
|  | Fatigue rating | Ave Fatigue Rating | Averaged over nights collected (score 0-10) | Daily sleep and activity log |
|  | Sleep rating | Ave Sleep Rating |  |  |
| Factors Affecting Sleep ^18, 19, 22-26^ | Alcohol use (in 6 hours prior to sleep) | Alcohol During Collection | Dichotomized to if it occurred over the collection period (no [0] or yes [1]) | Daily sleep and activity log |
|  | Caffeine use (in 6 hours prior to sleep) | Caffeine During Collection |  |  |
|  | Exercise | Exercised During Collection |  |  |
|  | Sleep medication use | Sleep Meds During Collection |  |  |
|  |  | PSQI: Sleep Meds | Use of over the counter or prescription medications for sleep from 0 (not during the past month) to 3 (3 or more times a week) | PSQI |
