# Supplementary material for: Limb accelerations during sleep are related to measures of strength, sensation, and spasticity among individuals with spinal cord injury
Source: J Neuroeng Rehabil. 2022 Nov 3;19:118. doi: 10.1186/s12984-022-01090-8 (PMC9635075; doi:10.1186/s12984-022-01090-8)
Supplement: Supplementary file 3 — Supplementary Material 3: Covariates Model Results_ESM.docx [file 12984_2022_1090_MOESM3_ESM.docx]

Supplementary Appendix 3: Strength (linear), sensation (linear), and spasticity (logistic) regression results for models built using only covariates and then with LA added to the covariate models.

| **Strength** | | | | | | | | | | | |  |
| --- | --- | --- | --- | --- | --- | --- | --- | --- | --- | --- | --- | --- |
| Feature Set | | Number of Features Selected | R^2^ | Adjusted R^2^ | $f$^2^ | | Mean Absolute Error | | Mean Squared Error | | Root Mean Squared Error |  |
| Covariates | | 19 | 0.740 | 0.492 | 0.97 | | 7.52 | | 77.75 | | 8.82 |  |
| LA + Covariates * | | 35 | 0.984 | 0.847 | 5.54 | | 1.68 | | 4.68 | | 2.16 |  |
| **Sensation** | | | | | | | | | | | |  |
| Feature Set | | Number of Features Selected | R^2^ | Adjusted R^2^ | $f$^2^ | | Mean Absolute Error | | Mean Squared Error | | Root Mean Squared Error |  |
| Covariates | | 2 | 0.262 | 0.222 | 0.29 | | 5.11 | | 36.83 | | 6.07 |  |
| LA + Covariates † | | 17 | 0.839 | 0.714 | 2.50 | | 2.03 | | 8.05 | | 2.84 |  |
| **Spasticity** | | | | | | | | | | | | |
| Feature Set | | Number of Features Selected | | F1-Score ‡ | | | Precision ‡ | | Recall ‡ | | OCA | |
| Covariates | | 5-6 | | 0.710 | | | 0.746 | | 0.711 | | 0.711 | |
| LA + Covariates § | | 12-16 | | 0.896 | | | 0.918 | | 0.895 | | 0.895 | |
| * adjusted R^2^_change_= 0.355 (72% increase), p= 0.021  † adjusted R^2^_change_= 0.492 (222% increase), p= 0.001  ‡ F1-score, precision, and recall represent the weighted average of the per-class scores.  § weighted average F1-score_change_= 0.186 (26% increase), no spasticity p< 0.001, mild spasticity p= 0.275, moderate spasticity p< 0.001 | | | | | | | | | | | | |
