# Supplementary material for: Limb accelerations during sleep are related to measures of strength, sensation, and spasticity among individuals with spinal cord injury
Source: J Neuroeng Rehabil. 2022 Nov 3;19:118. doi: 10.1186/s12984-022-01090-8 (PMC9635075; doi:10.1186/s12984-022-01090-8)
Supplement: Supplementary file 4 — Supplementary Material 4: LA Covariates Selected Features_ESM.docx [file 12984_2022_1090_MOESM4_ESM.docx]

Supplementary Appendix 4: LA features and covariates included in the strength and sensation linear regression and spasticity logistic regression models, sorted by the absolute value of the coefficient.

| **Strength**  (35 features) | | | | **Sensation**  (17 features) | | | | |  |  |
| --- | --- | --- | --- | --- | --- | --- | --- | --- | --- | --- |
| Feature Name | | | Coeff | Feature Name | | | Coeff | |  |  |
| Corr XY- IQR | | | 77.84 | Dom Freq 1- Med | | | -12.34 | |  |  |
| Angle Rate Change- Med | | | 71.32 | Power Dom Freq 2- Med | | | -10.42 | |  |  |
| Max Cross Cov- IQR | | | -69.68 | Time Since Prev- IQR | | | -10.34 | |  |  |
| Years Since Injury | | | 59.43 | Corr YZ- Med | | | 10.33 | |  |  |
| PSQI: Sleep Disturbance | | | -54.71 | Grav Change Y- IQR | | | 9.64 | |  |  |
| Ave Sleep Rating | | | 51.83 | Time Asleep | | | 8.95 | |  |  |
| Num Med Crossings Norm- Med | | | 49.94 | Wave Entropy- IQR | | | 8.06 | |  |  |
| Number if Pain Locations | | | -44.14 | Dom Freq 1- IQR | | | 7.94 | |  |  |
| Skewness- Med | | | 32.20 | Num Cross Cov Peaks- IQR | | | -6.29 | |  |  |
| PSQI: Poor Sleep Quality | | | 29.64 | PLM Index | | | 4.72 | |  |  |
| Med Freq- Med | | | -28.90 | Num Cross Corr Peaks- IQR | | | -4.07 | |  |  |
| PLM % | | | 28.51 | Number if Pain Locations | | | -3.43 | |  |  |
| Age | | | -27.69 | Power Dom Freq 1/Total- IQR | | | 2.72 | |  |  |
| Lyapunov Exp- IQR | | | 26.38 | Corr XZ- IQR | | | -2.42 | |  |  |
| Mean Freq- IQR | | | -24.91 | Mean Freq- IQR | | | 2.13 | |  |  |
| Move Next 90s- Med | | | -24.42 | Lyapunov Exp- Med | | | 1.52 | |  |  |
| PSQI: Sleep Meds | | | -23.91 | PSQI: Sleep Efficiency | | | -0.56 | |  |  |
| Num Cross Corr Peaks- IQR | | | 18.21 |  | | |  | |  |  |
| Wave Approx- IQR | | | 15.51 |  | | |  | |  |  |
| Sleep Meds During Collection | | | -12.67 |  | | |  | |  |  |
| Time Since Prev- IQR | | | -12.50 |  | | |  | |  |  |
| BMI | | | 11.57 |  | | |  | |  |  |
| PSQI: Sleep Duration | | | -10.74 |  | | |  | |  |  |
| Time Asleep | | | -9.58 |  | | |  | |  |  |
| PSQI: Sleep Latency | | | -8.62 |  | | |  | |  |  |
| Pain Interference: Sleep | | | -7.61 |  | | |  | |  |  |
| Alcohol During Collection | | | 6.62 |  | | |  | |  |  |
| Sex | | | 4.33 |  | | |  | |  |  |
| Caffeine During Collection | | | 3.96 |  | | |  | |  |  |
| SF-36: Pain | | | -3.38 |  | | |  | |  |  |
| Corr XY- Med | | | 2.21 |  | | |  | |  |  |
| Exercised During Collection | | | 1.96 |  | | |  | |  |  |
| Pain Present | | | 0.93 |  | | |  | |  |  |
| Power Dom Freq 1/Total- IQR | | | -0.82 |  | | |  | |  |  |
| PSQI: Sleep Efficiency | | | 0.77 |  | | |  | |  |  |
| **No Spasticity**  (15 features) | | **Mild Spasticity**  (12 features) | | | | **Moderate Spasticity**  (16 features) | | | | |
| Feature Name | Coeff | Feature Name | | | Coeff | Feature Name | | Coeff | | |
| PLM % | -2.08 | Time Asleep | | | 2.20 | Wave Approx- IQR | | -1.57 | | |
| Years Since Injury | 1.59 | Ave Fatigue Rating | | | 1.99 | Wave Entropy- IQR | | -1.56 | | |
| Wave Approx- IQR | 1.37 | Age | | | -1.83 | Corr YZ- Med | | 1.48 | | |
| Corr YZ- Med | -1.25 | Grav Change Z- IQR | | | -1.83 | Sleep Meds During Collection | | 1.30 | | |
| Skewness- IQR | 1.24 | Corr XY- Med | | | -1.28 | Skewness- IQR | | -1.29 | | |
| PSQI: Poor Sleep Quality | -1.03 | Power Dom Freq 1- IQR | | | 1.18 | Move/hour | | 1.28 | | |
| Power Dom Freq 2- Med | -1.02 | Num Cross Cov Peaks- IQR | | | -0.89 | Wave Energy 2- Med | | -1.25 | | |
| PSQI: Sleep Quality | 0.97 | Dom Freq Last 90s- IQR | | | -0.85 | Ave Sleep Rating | | -1.04 | | |
| Num Cross Cov Peaks- IQR | -0.91 | Dom Freq 1- Med | | | -0.71 | PSQI: Sleep Duration | | 1.01 | | |
| Sleep Meds During Collection | 0.79 | Exercised During Collection | | | -0.66 | PLM Index | | 1.00 | | |
| Dom Freq 2- IQR | 0.67 | Caffeine During Collection | | | 0.52 | PSQI: Sleep Disturbance | | -0.98 | | |
| Close Cross Cov Peak- IQR | 0.62 | PSQI: Sleep Meds | | | 0.14 | Dom Freq 1- Med | | 0.57 | | |
| Close Cross Corr Peak- IQR | 0.61 |  | | |  | Caffeine During Collection | | -0.50 | | |
| Wave Energy 2- Med | 0.58 |  | | |  | Med Freq- IQR | | 0.44 | | |
| Pain Interfere: Sleep | 0.47 |  | | |  | Med Freq- Med | | 0.41 | | |
|  |  |  | | |  | Pain Present | | 0.40 | | |
| Abbreviations: BMI= Body mass index, Coeff= Model Coefficient, Med= Median, PSQI= Pittsburgh Sleep Quality Index | | | | | | | | | |  |
| Covariates are in grey; LA features are in white. | | | | | | | | | |  |
